# Sustainability of weight loss from a family-centered pediatric weight management program integrated in primary care

**DOI:** 10.1186/s12913-021-07361-9

**Published:** 2022-01-02

**Authors:** Veronica Else, Qiaoling Chen, Alan B. Cortez, Corinna Koebnick

**Affiliations:** 1 Southern California Permanente Medical Group, Kaiser Permanente Yorba Linda Medical Offices, 22550 Savi Ranch Parkway, Yorba Linda, CA 92887 USA; 2 Department of Research & Evaluation, Kaiser Permanente Southern California, Pasadena, California USA; 3 Southern California Permanente Medical Group, Kaiser Permanente Tustin Ranch, Tustin, California USA

**Keywords:** Obesity, Weight management, Family-centered, Sustainability, Body mass index

## Abstract

**Background:**

A 6-month pediatric weight loss program showed modest success, but the sustainability of this success after 12 months was unclear. The present study aims tomeasure the medium-term effectiveness of family-based weight management in pediatric primary care to reduce body weight in children living with obesity.

**Methods:**

In a retrospective cohort study, children ages 3 to 17 years with obesity in Kaiser Permanente Orange County, California, who enrolled in a weight management program between April 2014 and December 2018 (FB-WMG, *n* = 341) were compared to children referred but not enrolled (Ref-CG, *n* = 317) and controls matched by sex, age, zip code and BMI (Area-CG, *n* = 801). The relative distance from the median BMI-for-age at months 0, 6, and 12 were expressed as difference-in-differences (DID) using multivariable linear regressions with robust standard error.

**Results:**

The baseline BMI-for-age was 98.6 (SD 1.08) percentile in FB-WMG, 98.2 (SD 1.22) percentile in Ref-CG, and 98.6 (1.13 in Area-CG). FB-WMG had a median of 3 visits (P25 1 visit, P75 5 visits) in the first 6 months. Despite a more considerable decrease in the relative distance to the median BMI-for-age in FB-WMG children with 3+ visits after 6 months, the success obtained was not sustained at 12 months (DID FB-WMG vs Area-CG -0.34, 95% CI − 3.00 to 2.33%, FB-WMG vs Ref-CG -0.39, 95% CI − 3.14 to 2.35%). At 12 months, there was no statistical significant difference between the three groups (FB-WWG, Ref-CG, Area-CG).

**Conclusions:**

The initial success in weight management was not sustained in the absence of continued support for healthy lifestyle changes. Based on current evidence, continued support is necessary to maintain and promote success beyond a brief 6 month intervention. Long-term pediatric weight management programs are needed to promote continuing progress.

## Introduction

In the United States during 2015-2018, 19% of 6 to 11 year old children and 21% of adolescents between 12 and 19 years of age were living with obesity [1]. Obesity in adults increases the risk of metabolic disease and is associated with premature death [2–4]. The estimated annual obesity-related healthcare costs in the U.S. are $190 billion or 21% of the annual medical spending, with childhood obesity alone costing $14 billion [5]. Preventing and treating obesity is more important than ever in the light of the COVID-19 pandemic and related measures such as school closures and stay-at-home orders. Obesity increases the risk of severe forms and death from COVID-1 9[6]. Youth ages 5 to 17 years gained more weight during the COVID lockdown than before the pandemic, resulting in an increase in obesity by 5 %[7].

Obesity in adolescents is highly predictive of obesity during adulthood, with over 85% of obese adolescents ages 15-17 growing up to be obese at age 50 years [8]. It is crucial to prioritize the prevention and early treatment of childhood obesity [9–12]. Unfortunately, low or moderate dose weight management intervention has demonstrated little or unclear benefits [13]. The U.S. Preventive Services Task Force (USPSTF) concluded that comprehensive, intensive behavioral interventions with a total of 26 contact hours or more are required to achieve sustained weight loss [14]. However, this approach is hampered by limited available resources, high attrition of participants, and weight gain relapses [15–18].

This study is a follow-up to existing weight management program, a comprehensive behavioral lifestyle-change intervention with less contact hours as recommended by the USPSTF. During the first 6-months of the program, participants decreased their body mass index (BMI) by 0.85 kg/m^2^ after 6 months compared to a control group after participating in a low to moderate dose family-based weight management program in a primary care setting [19]. The intervention was designed as a 4 to 6-month program with optional on-demand visits between 6 and 12 months. In our current study, we evaluate the effectiveness of the program after 1-year of follow-up.

## Methods

### Study design, setting, and population

**Fig. 1:**
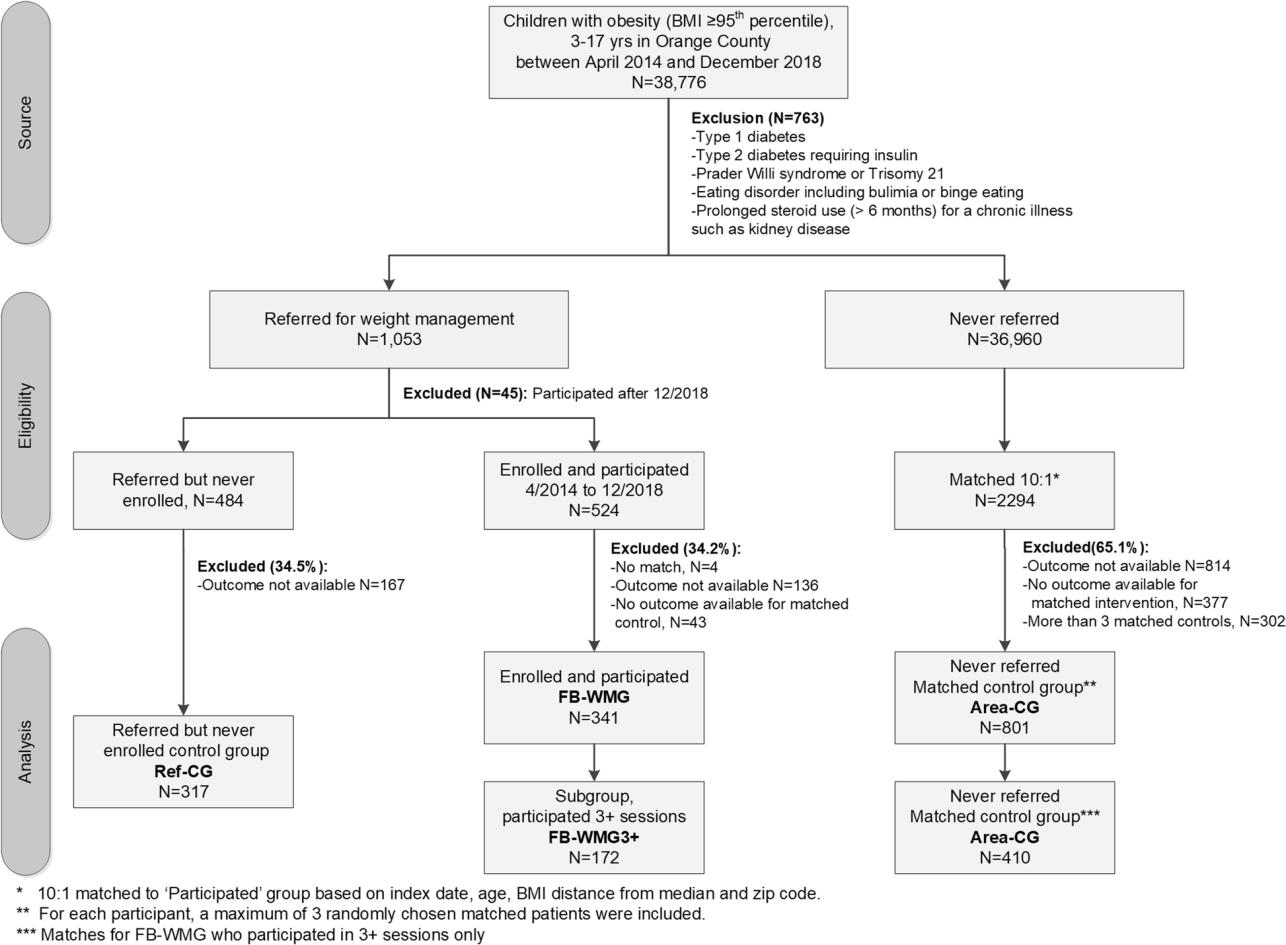
Study flow chart

For this retrospective cohort study, we identified youth (*n* = 38,776) with obesity (defined as BMI-for-age ≥ 95th percentile) [20] between the ages of 3 and 17 years who received care at the Kaiser Permanente Medical Offices in Orange County, California, between April 2014 and Dec 31, 2018 (Fig. 1). The study cohort was described in detail elsewhere [19]. The study protocol was reviewed and approved by the KPSC Institutional Review Board. Informed consent was waived for this study.

Youth were eligible for the study if they were obese (BMI-for-age ≥ 95th percentile), did not have insulin-dependent diabetes, chromosomal anomalies predisposing to obesity such as Prader-Willi syndrome or Trisomy 21, eating disorders including bulimia or binge eating, prior history of medications or surgery for the treatment of obesity, did not have a history of prolonged steroid use (> 6 months) for treatment of a chronic illness, and were not pregnant.

For the intervention group, we identified youth who were enrolled in a family-based weight management program using International Classification of Diseases, 9th and 10th Revision, Clinical Modification (ICD-9 and ICD-10) Z71.3 and V65.3 and a KPSC-specific code for weight management. We included youth participating in one or more sessions over 12 months of follow-up (*n* = 341) to assess a dose-response relationship (Fig. 1).

As control groups, we identified youth with obesity who were referred to the family-based weight management program but did not participate in any sessions (Ref-CG, *n* = 317), as well as area-matched youth who were never referred and were not enrolled in a K.P. weight management program (Area-CG, *n* = 801).

### Family-based weight management intervention

The Orange Country pediatric weight management program started in 2014 to support families in managing their child’s body weight. Individual appointments provided the ability to identify specific needs and barriers to change. Four pediatric care providers administered the family-based weight management program (FB-WM). The program used motivational interviewing (MI) approaches [21–26]. Youth were referred to the program by their care provider. The program consisted of 30 min counseling appointments provided by a pediatric nurse practitioner or pediatrician. While many counseling models rely heavily on directive advice and information exchange, an MI counselor generally avoids direct attempts to convince or persuade. MI is a patient-centered counseling style that explores, strengthens, and guides an individual’s motivation for change [27, 28]. It relies on specific techniques, which include reflective listening and eliciting change talk [29, 30]. The visit included the child and usually one or more parent or caregivers; in some cases, the entire family. Family members accompanying the child varied from visit to visit. About 1/3 of visits with adolescent patients took place without the presence of a parent or guardian by choice. In these cases, parents were contacted by phone to ensure family involvement. These visits were offered to provide patient-centered care and to avoid exclusion of teenagers with working parents. The visits focused on one or more of the following target areas: snack foods, sweetened beverages, eating out, whole grains, fruits, vegetables, sweets and desserts, portion size, T.V./screen time, video games, and physical activity. Provider and patient determined together which behaviors were most amenable to intervention, which goal they wanted to commit to, and work into a personalized plan. This meal/exercise plan was created and provided for each patient/family. Handouts developed by Kaiser Permanente for weight management supported the goal setting. If needed, we sent letters to schools, babysitters, and other caretakers asking for their help supporting the family. The core of the intervention used the 5-4-3-2-1-GO! Tool developed by the Consortium to Lower Obesity in Chicago Children (CLOCC) [31–33]. This plan implemented 5 fruits/vegetables per day, 4 glasses of water daily, 3-servings of low-fat dairy daily (alternatives provided for children with lactose intolerance or those who refuse dairy), 2 h or less of screen time per day, 1 h physical activity per day, and 0 sugared drinks. The healthy plate materials were adopted [34]. These guidelines were reviewed at each visit and used to review progress, provide feedback, and support goal setting.

A written food diary was reviewed at follow-up visits, and if not available, was obtained by memory recall. Encouragement was given for positive changes, regardless of a weight change. Provider and patient identified problem areas when applicable and discussed solutions. Behavioral goals and BMI changes were reviewed if appropriate.

The program was designed to be completed in 3 to 6 thirty minute counseling sessions (1.5 to 3 h). Patient, family, and care providers decided together when the intervention was complete but had the option to continue if additional counseling sessions were needed, even beyond the original length of the 6-month program. In the present analysis, all patients were included as “intention-to-treat” even if the patient/family discontinued the program before the third visit.

Efforts to train all providers in MI techniques are ongoing and not fully implemented. Informal training has been provided to many but not all pediatric care providers, and they are currently not certified in MI with standardized quality controls and regular training. The same four providers consistently provided care. The fidelity of the intervention based on a written framework was maintained through regular team meetings but without formal fidelity assessments.

### Intervention and control groups

The analytic cohort consists of three groups: 1) a group of children who received a family-based behavior-changing weight management intervention (FB-WMG), 2) a control group of children referred by their provider who did not participate in the intervention (Ref-CG), and an area control group (Area-CG) matched for sex, age, relative distance from the median BMI-for-age, zip code and time of the first visit (allowing +/− 1 year around the window of their match). We used a 2-step matching approach. In the first step, we matched 230 out of 341 children using index date (+/− 365 days), age (+/− 1 year), relative distance from the median BMI-for-age (+/− 0.5%), and exact zip code. During the second step, another 111 children were matched using index date (+/− 548 days), age (+/− 1 year), and relative distance from the median BMI-for-age (+/− 0.5%). The index date for FB-WMG was at the first appointment. For Ref-CG, the referral date (or nearest office visit date with BMI) was used as the index date. For Area-CG, the index date was the date of the BMI measure used for matching.

Height and weight were measured in light clothing without shoes and used to calculate sex-specific BMI-for-age [20]. To determine BMI at 6 months of follow-up, BMI was calculated from weight and height measured during a routine outpatient visit within 1 month before and 3 months after 6 month of follow-up. If no weight and height were available close to the 6-month follow-up, BMI was calculated by linear regression using 2 weights and heights within 3 months around the 6-month follow-up (< 10% of measures). To determine BMI at 12 months of follow-up, BMI was calculated from weight and height measured during a routine outpatient visit closest to the 12 months follow-up within 3 months before and 3 months after 1-year follow-up. If no weight and height were available close to the 12 months of follow-up, BMI was calculated by linear regression using 2 weights and heights within 6 months around the 12-month follow-up (< 5% of measures).

### Study outcome

Change in body weight was calculated for each follow-up visit as the absolute and relative difference in the distance from the median BMI-for-age and sex [35]. This metric is a more reliable measure of change in adiposity, particularly for individuals in the upper end of the BMI distribution compared to other methods such as BMI z score [35]. It also does not have an upper limit (as has BMI-for-age percentile) and can be used to assess adiposity across the entire BMI spectrum.

### Covariates

We obtained race and ethnicity information from health plan administrative records and birth records. We categorized race/ethnicity as non-Hispanic White, Hispanic (regardless of race), African American, Asian or Pacific Islander, and other, multiple or unknown race/ethnicity. In addition, we used median household income and education in the patient’s residential census tract as area-based measures of socioeconomic status [36, 37]. Census-tract household income was classified using the individual’s likelihood of a median household income of < $45,000, $45,001 to $80,000, $80,000 or more. Neighborhood education was categorized using an individual’s likelihood of education with some college or higher. We also used insurance through government healthcare assistance programs (yes/no), such as MediCal, as an additional proxy for socioeconomic status.

### Statistical analysis

Baseline characteristics of the study cohort are presented for all three study groups: for the family-based behavior-changing weight management intervention group (FB-WMG), referred control group (Ref-CG), and area control group (Area-CG) using means with standard deviation (S.D.) or medians with interquartile range (P25 – P75) for continuous variables as appropriate, and the number of observations with percentage for categorical variables. Differences in characteristics between groups are assessed using the t-test, chi-squared test or fisher’s exact test contrasting intervention against control groups.

We conducted sensitivity analyses restricting FB-WMG youth to those with at least three visits and their matched Area-CG. The primary outcome measure was a change in the relative distance from the median BMI-for-age between baseline and 12-month follow-up. In addition, we present outcomes of the 6-month follow-up. Adjusted differences-in-difference (DID) and confidence intervals in relative distance from the median BMI-for-age between intervention and control groups are derived using multivariable linear regressions with robust standard error.

All models are adjusted for age, sex, race/ethnicity, baseline BMI, state-subsidized insurance coverage, neighborhood income and education, and length of KPSC membership. For simplicity and consistency, we included all three study groups in one model. We performed additional analyses using separate mixed linear regression to compare FB-WMG and Area-CG to account for the matching process. The results were essentially consistent and did not affect the overall conclusion. All analyses were performed using SAS statistical software version 9.4 (SAS Institute Inc., Cary, NC).

## Results

The majority of youth participating in the FB-WMG (*n* = 341) were 9 years and older (*n* = 268, 78.6%) and Hispanic (*n* = 234,68.6%). FB-WMG youth were similar to youth in the matched Area-CG (*n* = 801), and Ref-CG with respect to age, sex, and state-subsidized health insurance and but FB-WMG were from neighborhoods with higher median household income and neighborhood education (Table 1). The distance from the median BMI-for-age at baseline adjusted for the mean cohort age (11.2 years) was ~ 12.5 to 12.8 kg/m^2^ and similar across all groups. A subgroup of 172 out of 341 youth (50.4%) in the FB-WMG received 3 or more counseling sessions (Table 1).

**Table 1:** Baseline characteristics of children of the family-based weight management group (FB-WMG), matched area control group (Area-CG), referred but untreated control group (Ref-CG)

	Study group				***P***-Value	
	FB-WMG					
	All (1+ session)	With 3+ sessions	Area-CG	Ref-CG	FB-WMG* vs. Area-CG	FB-WMG* vs. Ref-CG
**N**	341	172	801	317		
**Age (years)**, N (%)					0.770	0.645
3-8	73 (21.4)	56 (32.6)	169 (21.1)	80 (25.2)		
9-12	151 (44.3)	41 (23.8)	360 (44.9)	123 (38.8)		
13-17	117 (34.3)	75 (43.6)	272 (34.0)	114 (36.0)		
**Sex**, N (%)					0.939	< 0.001
Girls	169 (49.6)	98 (57)	395 (49.3)	113 (35.6)		
Boys	172 (50.4)	74 (43)	406 (50.7)	204 (64.4)		
**Race/ethnicity**, N (%)					0.029	0.074
White	65 (19.1)	29 (16.9)	152 (19)	70 (22.1)		
Black	6 (1.8)	5 (2.9)	30 (3.7)	10 (3.2)		
Hispanic	234 (68.6)	119 (69.2)	535 (66.8)	206 (65)		
Asian/Pacific Islander	33 (9.7)	19 (11)	57 (7.1)	21 (6.6)		
Other/Multiple	3 (0.9)	0 (0)	27 (3.4)	10 (3.2)		
**BMI-for-age percentile**						
Mean (SD)	98.6 (1.08)	98.7 (1.01)	98.6 (1.13)	98.2 (1.22)	0.752	< 0.001
**Absolute distance from median BMI-for-age (kg/m**^**2**^**)#**						
Mean (SD)	13.8 (4.90)	13.9 (4.75)	13.7 (4.89)	13.2 (5.07)	0.759	0.090
**Relative distance from median BMI-for-age (%)#**						
Mean (SD)	77.9 (27.71)	78.4 (26.83)	77.4 (27.68)	74.7 (28.62	0.756	0.104
**State-subsidized insurance**, N (%)					0.15	0.53
0	168 (49.3)	80 (46.5)	432 (53.9)	164 (51.7)		
1	173 (50.7)	92 (53.5)	369 (46.1)	153 (48.3)		
**KPSC membership duration (years)**						
Mean (SD)	6.3 (4.13)	6.6 (4.07)	5.8 (4.27)	5.4 (4.17)	0.026	0.002
**Neighborhood education** (% college degree and higher)					0.013	0.028
0-50%	132 (38.7)	66 (38.4)	366 (45.7)	142 (44.8)		
51-75%	121 (35.5)	59 (34.3)	290 (36.2)	120 (37.9)		
76-100%	88 (25.8)	47 (27.3)	145 (18.1)	55 (17.4)		
**Neighborhood median household income**					0.019	0.089
< =$45,000	54 (15.8)	23 (13.4)	146 (18.2)	45 (14.2)		
$45,001-$80,000	154 (45.2)	72 (41.9)	411 (51.3)	170 (53.6)		
> $80,000	133 (39.0)	77 (44.8)	244 (30.5)	102 (32.2)		

#Adjusted for the mean age of the cohort (11.5 years)

*Comparison to total FB-WMG (*n* = 341)

**Fig. 2:**
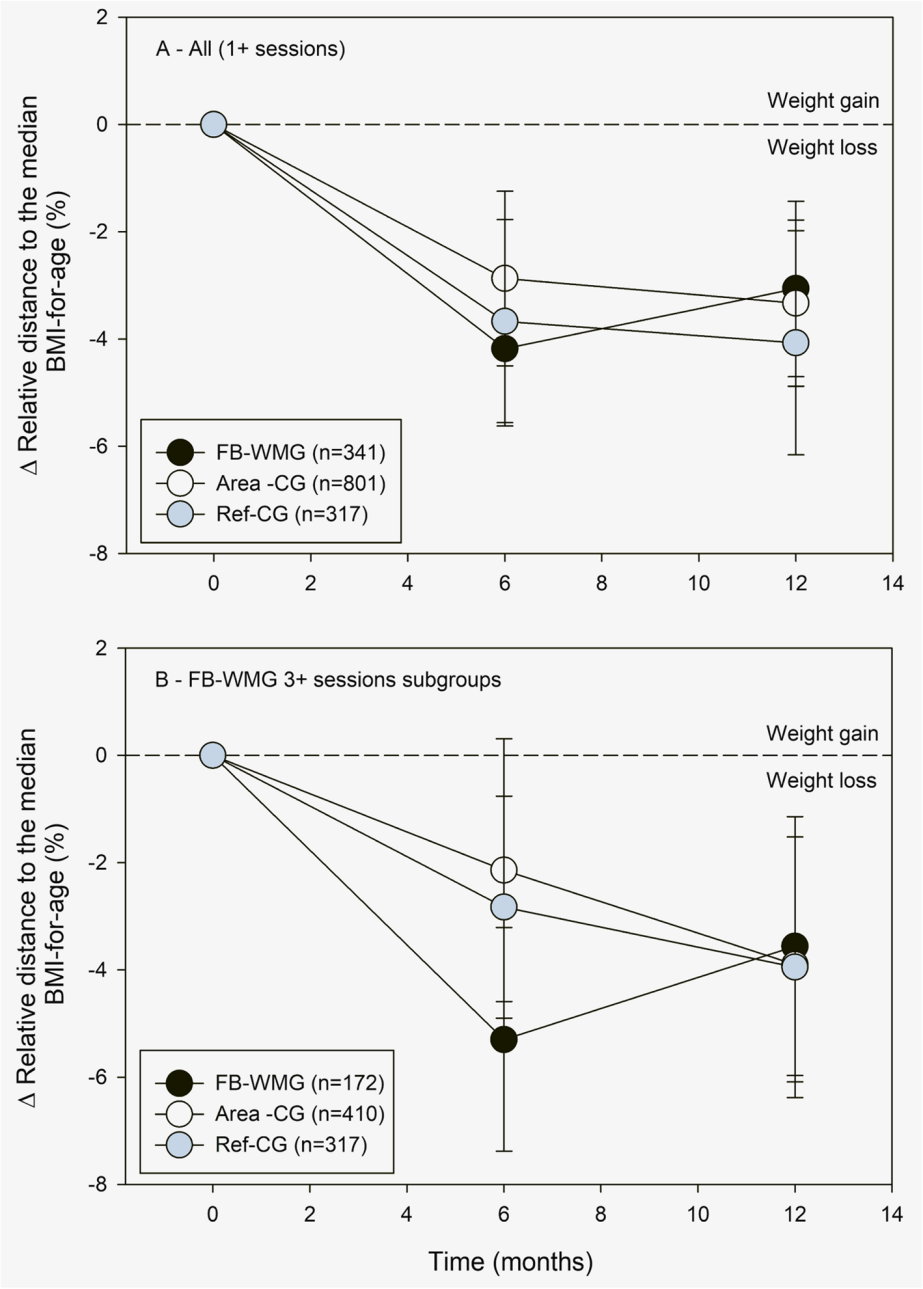
Change in relative distance to the median BMI-for-age from baseline to 6 and 12 months in children of the family-based weight management group (FB-WMG) with at least one counselling session (**a**) and with 3 or more counselling sessions (**b**), their matched area control group (Area-CG), referred but untreated control group (Ref-CG)

**Fig. 3:**
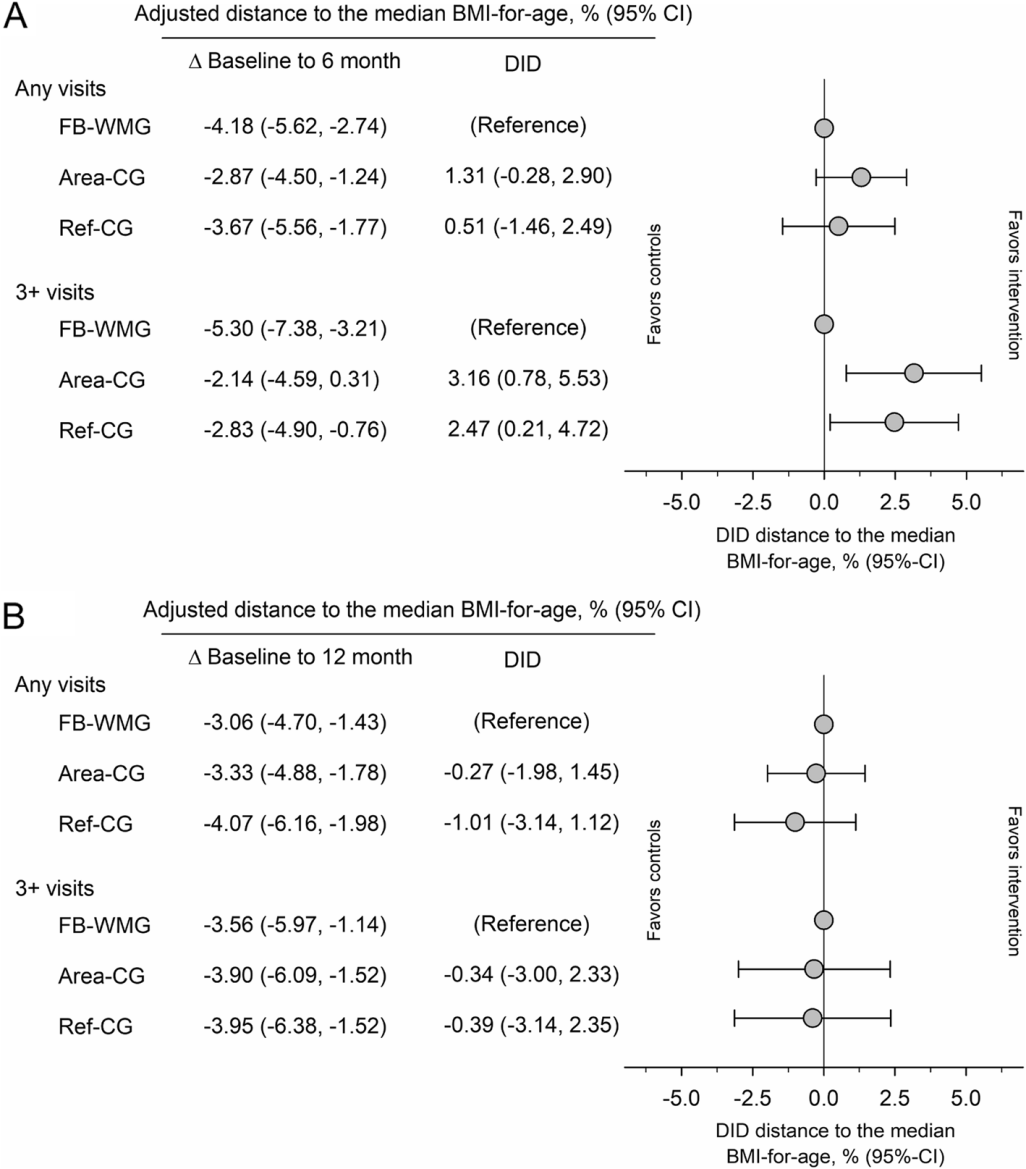
Change in the distance to the median BMI-for-age and difference-in differences (DID) in children of the family-based weight management group (FB-WMG), matched area control group (Area-CG), referred but untreated control group (Ref-CG) after 6 months (**A**) and after 12 months (**B**)

After 6 months, FB-WMG youth, including those who received only one counseling session, decreased their distance to the median BMI-for-age by − 4.18% (95%CI – 5.62 to − 2.74%), adjusted for sex, race, state-subsidized health insurance, membership duration, neighborhood income and education (Fig. 2a). When restricting to FB-WMG youth who received 3 or more counseling sessions (Fig. 2b), the adjusted distance to the median BMI-for-age decreased by − 5.30% (95%CI − 7.38 to − 3.21%). For youth with at least one counseling session, the difference-in-differences (DID) between FB-WMG and Area-CG was 1.31% (95% CI − 0.28 to 2.90%) and between FB-WMG and Ref-CG 0.51% (95% CI − 1.46 to 2.49). (Fig. 3a). For youth who received 3 or more counseling sessions, the DID between FB-WMG and Area-CG was 3.16% (95% CI 0.78 to 5.53%) and between FB-WMG and Ref-CG 2.47% (95% CI 0.21 to 4.72%, Fig. 3b).

The 12-months follow-up data indicate that the weight loss in FB-WMG was not sustained compared to the other groups. FB-WMG youth, including those who received only one counseling session, had a decrease in the adjusted distance to the median BMI-for-age compared to their baseline of − 3.06% (95%CI – 4.70 to − 1.43%). When restricting to FB-WMG youth receiving 3 or more counseling sessions (Fig. 2), the distance to the median BMI-for-age compared to their baseline was − 3.56% (95%CI − 5.97 to − 1.14%). For youth with at least one counseling session, the difference-in-differences (DID) between FB-WMG and Area-CG was − 0.27% (95% CI − 1.98 to 1.45%) and between FB-WMG and Ref-CG -1.01% (95% CI − 3.14 to 1.12) (Fig. 3a). For youth who received 3 or more counseling sessions, the DID between FB-WMG and Area-CG was − 0.34% (95% CI − 3.00 to 2.33%) and between FB-WMG and Ref-CG -0.39% (95% CI − 3.14 to 2.35%, Fig. 3b).

We were not able to identify any subgroups with sustained success across groups defined by sex, race/ethnicity, neighborhood income, neighborhood education, and SES. Additional unstructured and incidental visits after the initial intervention of 6 months also did not affect the medium-term effect of the intervention.

## Discussion

The present study is a continued evaluation of a low to moderate intensity family-based weight management intervention, including a pediatric provider in primary care in which 70% of youth reduced or maintained BMI after only 6 months compared to 45 and 58% in the two control groups [19]. Here, we investigated if the program’s success could be sustained 12 months after entering the program. Our analyses show that the 6-months weight loss of our low to moderate intensity 6-month program with 3+ counseling sessions was not sustained without a structured follow-up program.

The family-based weight-management evaluated here with a median of 3 counseling sessions (equivalent to 1.5 h) and 5 visits for the highest quartile (equivalent to 2.5 h) was significantly below the 26 h of comprehensive lifestyle intervention recommended by the USPSTF for sustained long-term change [14, 38]. Early attrition could have contributed to lower weight loss and stabilization because most families ended the program after 3 visits. Families may have been over-confident in their ability to continue their weight management on their own, or disliked the financial or time burden from additional appointments.

A meta-analysis of 20 behavioral family lifestyle interventions for childhood obesity with a follow-up of 10 to 24 months suggested a small effect on BMI. However, the dose of treatment - measured by the number of intervention sessions and number of minutes spent in treatment - was positively related to effectiveness [39]. Higher intervention intensity was associated with better outcomes [40]. The present study has moderate success for those with > 3 or more counseling sessions over 6 months (≥ 1.5 h), but the effect was not sustained at 1 year, independent of additional unstructured visits. Thus, our data indicate that further intervention beyond 6 months with additional counseling is needed for sustained outcomes.

The difference in weight change between intervention and two control groups was not significant at 12 months from intervention, with all three groups showing similar weight loss. The weight loss observed in the control groups may be explained by additional educational tools and weight management resources outside of the FB-WM program evaluated here. All providers were trained to identify high-risk patients with obesity and support families to promote change regardless of a referral to the FB-WM program or the patient seeking active treatment [41, 42]. Hence, youth in the Ref-CG or Area-CG may have received resources that were unaccounted for in our present analysis.

Stabilization of BMI in children can be achieved through family-based pediatric weight management programs, but realistic goals must be addressed among families and professional caregivers [43]. A randomized trial of obese youth ages 5 to 11 years measured BMI z scores after 6 months of lifestyle changes focused on fitness at a recreation sports facility. Although BMI z scores did not change significantly, improvements in physical activity and quality of life were noted in the intervention group [44]. In all pediatric weight management interventions, lifestyle changes may contribute to improved health without reducing weight or BMI [14].

School and community-based programs and medical clinic-based interventions or referral may help eliminate specific barriers to care compared to interventions provided by the medical community alone. These barriers include the effort to travel to medical appointments and share of cost (co-pay). One meta-analysis of 139 intervention studies, of which 83% were school-based, indicated that school-based interventions combined with home involvement had the highest proportions of studies with favorable results [45].

Another barrier to weight management programs was time effort. Prior to participation in our family-based pediatric weight management program, the provider asked parents to commit to a 4 to 6 months program, approximately one visit per month, until a mutual agreement about the discharge from the program. More frequent visits may have been beneficial but not possible due to limited staffing resources. In our study, 47% of participants left the program before the completion of 3 counseling sessions. Reasons for attrition included unreadiness to change, scheduling or transportation issues, and share of program and visit costs. A study investigating attrition among pediatric weight management programs across three Canadian sites showed 83% discontinued care early due to a range of multi-level factors, including perceived lack of progress, lack of motivation, family support, logistical factors, cost, and unmet care expectations [17]. Across 29 pediatric obesity programs, up to 73% of participants drop out early and cited unrealistic expectations and lack of motivation as significant contributors [17]. The authors [17] felt that clinician family bonding could help with expectations and address value of treatment, and added that the focus of care should be on health and habits, and not weight or a number on the scale [18].

### Study limitations

Since treatment by our program was not randomized, unmeasured confounders may have limited the ability to measure differences among the groups. Unaccounted factors may include underlying behavior issues, lack of motivation or other unmeasured limitations, and the ability for some families in control groups to obtain outside resources or programs. On the other hand, the results of this pragmatic observational study reflected a real-world environment and allowed us to evaluate the effects in such settings without highly selective inclusion and exclusion criteria. The intervention group was also self-selected. They may have participated because other treatment programs have failed before, because of a fear of disease, a desire to please the referring physician, or other unknown reasons. Moreover, our intervention group who participated in at least 3 sessions was relatively small (*n* = 172), making it challenging to identify subpopulations that best responded to treatment.

In conclusion, our family-based low-to-moderate intensity weight management program may have been hampered by its low intensity and the lack of a longer follow-up to sustain initial success and better medium-term outcomes. Considering the long-term health consequences of obesity, childhood obesity intervention programs should be a healthcare priority. More resources are necessary toreduce the prevalence of excess weight among children. .

## Data Availability

The datasets generated and analyzed during the current study supporting the findings of this study are not publicly available due to privacy restrictions but are available from the corresponding author upon reasonable request and with written permission of the Kaiser Regional Research Committee of Kaiser Permanente Southern California (contact through corresponding author, https://www.kp-scalresearch.org/aboutus/physician-research-support/regional-research-committee/).

## Abbreviations

BMI: Body Mass Index
K.P.: Kaiser Permanente
PCP: Primary Care Provider
B/P: Blood pressure
FB-WMG: Family based weight management
Ref-CG: Referred control group
Area-CG: Area controlled group.
